# Experience of physical violence during pregnancy and its association with behavioral outcomes during the prenatal and postnatal period: a pooled analysis of cross-sectional data from 45 low-income and middle-income countries

**DOI:** 10.1016/j.eclinm.2025.103491

**Published:** 2025-09-19

**Authors:** Joyce Agbo, Davis Amani, Neema Mosha, Heidi Stöckl

**Affiliations:** a Institute for Medical Information Processing, Biometry and Epidemiology, Faculty of Medicine, Ludwig-Maximilians-University of Munich, 81377, Munich, Germany; b Pettenkofer School of Public Health, Munich, Germany; c Mwanza Intervention Trials Unit, Mwanza, Tanzania

**Keywords:** Intimate partner violence (IPV), Pregnancy, Maternal health, Low-income and middle-income countries (LMICs), Antenatal care, Demographic and health surveys (DHS)

## Abstract

**Background:**

Although previous studies have established a link between physical intimate partner violence (IPV) and adverse health outcomes for mothers and children, there is a lack of thorough comparative analysis in low-income and middle-income countries (LMICs) that examines how physical IPV experienced during pregnancy specifically differs from physical IPV at other times. This comparison is crucial to understanding the extensive impact of physical IPV during pregnancy on antenatal care, early initiation of breastfeeding (EIBF), exclusive breastfeeding for the first two days after birth (EBF2D), and place of delivery (POD).

**Methods:**

This study conducted secondary analyses using cross-sectional data from the Demographic and Health Surveys (DHS) of 45 LMICs collected between 2012 and 2022 which utilized a two-stage stratified sampling method to include women who were interviewed for the domestic violence module and had a child 2 years old or younger. Multivariable log-binomial regression models were utilized to examine the associations between IPV, both during pregnancy and at other times, and the specified outcomes.

**Findings:**

After applying survey weights, 100,199 women were included in the analyses. The prevalence of physical IPV during pregnancy was 6.07% (n = 6078). Adjusted for covariates, physical IPV during pregnancy was negatively associated with adequate antenatal care utilization (RR = 0.88; 95% CI = 0.84, 0.91), EIBF (RR = 0.92; 95% CI = 0.89, 0.96), and EBF2D (RR = 0.93; 95% CI = 0.90, 0.96). While physical IPV at other times also negatively impacted most outcomes, the effect was more pronounced when physical IPV occurred during pregnancy.

**Interpretation:**

Physical IPV both during pregnancy and at other times pose significant barriers to maternal healthcare utilization and optimal breastfeeding practices in LMICs, with the impact of physical IPV during pregnancy being more severe. Targeted antenatal care interventions addressing physical IPV during pregnancy could improve health outcomes for both mothers and children.

**Funding:**

The manuscript was supported by the European Research Council Consolidator Grant IMPROVE_LIFE (Grant number 101124718).

**Research in context**

**Evidence before this study**

Databases like PubMed, Google Scholar, and JSTOR were searched. Additionally, the Demographic and Health Surveys (DHS) database was consulted for relevant data. Search terms used include: “intimate partner violence,” “IPV,” “pregnancy,” “maternal health,” “antenatal care,” “facility-based delivery,” “low and middle-income countries (LMICs)”, “breastfeeding practices,” “postnatal care,” “skilled birth attendance,” “health outcomes,” “demographic health surveys.” Many relevant studies were identified including systematic reviews and meta-analysis. Four relevant studies which included multi-country analysis, scoping review, systematic review, and meta-analysis were identified. The first study conducted by Karen Devries and colleagues based on data from 19 countries, demonstrated that the prevalence of intimate partner violence (IPV) during pregnancy ranged from 2.0% in Australia to 13.5% in Uganda with the prevalence being higher in African and Latin American countries. The second, a scoping review of 26 studies from 13 LMICs conducted by Thao Da Thi Tran and colleagues, suggested that IPV during pregnancy was significantly associated with higher odds of postpartum depression, less breastfeeding, and low birth weight. The third study from Abdulbasit Musa and colleagues was a systematic review and meta-analysis that looked at the association between IPV and maternal health care service utilization and demonstrated that women who experienced IPV had reduced odds of adequate antenatal care utilization and skilled delivery care compared to those who did not experience IPV. The fourth study is a scoping review of 16 studies from 10 LMICs by Methany and Stephenson which looked at the association of IPV and the uptake of antenatal care. IPV experience was negatively associated with the initiation of antenatal care and several visits.

**Added Value of this Study**

This study significantly advances our understanding of the impacts of IPV by distinguishing between IPV during pregnancy and IPV at other times. Pregnancy represents a period of increased physical and emotional vulnerability for women. The stress and physical demands of pregnancy, combined with the experience of IPV, may lead to more severe health outcomes for both the mother and the child compared to IPV experienced at other times. By using a large, representative sample from multiple LMICs, this study ensures the generalizability of its findings across different regions and settings.

**Implications of all the available evidence**

This research not only reinforces the need for targeted (antenatal care) interventions to support pregnant women experiencing IPV but also highlights the importance of integrating IPV education and prevention programs into maternal health services to mitigate these adverse effects. Policies should focus on training healthcare providers to recognize signs of IPV during antenatal care visits and provide targeted interventions. For further research, cohort studies should be considered to explore the long-term effects of IPV during pregnancy on child development and maternal health. Also, qualitative studies could provide deeper insights into experiences of IPV during pregnancy and the effectiveness of intervention strategies.

## Introduction

Intimate partner violence (IPV) is a significant public health concern, with physical and sexual IPV affecting nearly one-third of women globally (27%).^1^ IPV can have severe health consequences for both the mother and the unborn child.^2^ Previous studies indicate that IPV during pregnancy is also widespread, with its prevalence varying significantly across different regions globally, with a range of up to 13.5%.^3^ IPV during pregnancy has also been linked to adverse physical and mental health outcomes for women, including physical injuries, depression, anxiety, and poor maternal and perinatal health.^4^, ^5^, ^6^ Additionally, pregnant women experiencing IPV are more likely to have reduced utilization of antenatal care, not adhere to the recommended breastfeeding practices, and have a lower likelihood of delivering in a healthcare facility.^6^^,^^7^ Poor partner relationships during the period of pregnancy can trigger chronic stress responses that could increase the risk of adverse outcomes such as low birth weight and preterm birth.^8^

Antenatal care is a crucial component to ensure maternal and child health, through the ongoing monitoring of the health of pregnant women and the fetus, as well as providing education on maternal and newborn care. Inadequate antenatal care can lead to pregnancy complications and significantly increase the likelihood of maternal or perinatal mortality.^9^ The global maternal mortality ratio remains significantly high (223 maternal deaths per 100,000 live births), with the greatest risk concentrated in low-income and middle-income countries (LMICs).^10^

IPV during pregnancy can act as a significant barrier to accessing and utilizing antenatal care services. A meta-analysis revealed that women who experienced IPV had a 25% reduced likelihood of utilizing antenatal care.^11^ This negative association may be attributed to controlling behaviors and power imbalances inherent in abusive relationships, which can limit a woman's autonomy, freedom of movement, and decision-making ability, hindering her access to antenatal care.^12^^,^^13^ Key factors associated with both antenatal care utilization and IPV in pregnancy, particularly in LMICs, are education, wealth index, employment status, age, parity, decision-making autonomy, place of residence, mass media exposure, and accessibility of health facilities.^4^^,^^12^

Early initiation of breastfeeding (EIBF), defined as putting the infant to the breast within 1 h of birth, and exclusive breastfeeding for the first two days after birth (EBF2D), are crucial for both maternal and child health.^14^ Benefits of EBIF and EBF2D include early skin-to-skin contact and suckling, which trigger hormonal responses that facilitate milk production. Colostrum, the first milk, is rich in antibodies and nutrients critical for the newborn.^15^, ^16^, ^17^ Early suckling further facilitates uterine contractions and reduces postpartum bleeding. A study examining data from 51 LMICs found that mothers exposed to any form of IPV had a 12% lower likelihood of EIBF, after adjusting for the three forms of IPV, only physical IPV remained statistically significant.^10^ The negative association between IPV and early breastfeeding initiation remains consistent across different regions and contexts, although the strength of the association varies.^18^^,^^19^ Past research consistently highlights that socioeconomic disadvantages, lack of empowerment, reproductive health issues, and limited access to healthcare services are common risk factors that can lead to both inadequate breastfeeding practices and increased vulnerability to IPV during pregnancy.^18^, ^19^, ^20^

Delivering in a health facility is crucial for the health and survival of both the mother and the newborn baby, as it allows skilled health care providers to identify and handle complications that may arise during labour and childbirth.^21^ Based on previous studies, there is limited direct evidence examining the association between physical IPV during pregnancy and the place of delivery (POD), i.e., home vs. health facility. A study from Bangladesh found that women who experienced IPV were significantly less likely to receive delivery care from a medical professional.^22^

IPV during pregnancy, particularly physical violence, is considered a severe form of IPV with significant negative consequences for both the mother and the child. While psychological and emotional abuse alone can have detrimental effects, the presence of physical violence during pregnancy is considered a marker of severe and high-risk IPV.^23^ Physical IPV is rarely experienced in isolation. It often co-occurs with emotional, sexual, or economic abuse, and can serve as a marker for broader patterns of partner control and coercion.^24^ By using physical IPV as a measurable and severe indicator, our study highlights a clearly defined and policy-relevant form of violence, while remaining grounded in a broader understanding of IPV as a continuum of harm that deserves further exploration in future research. However, the occurrence of IPV during pregnancy itself is rarely considered in studies that included multiple LMICs on the effect of IPV on antenatal care, early breast feeding and POD, although IPV during pregnancy is more closely associated with the prenatal period and immediate postnatal outcomes and hence likely to have a stronger impact on how mothers act and seek healthcare. Given these considerations, there is a pressing need for a multi-country analysis that specifically compares the effects of physical IPV during pregnancy, physical IPV at other times, and no experience of physical IPV on key maternal and child health outcomes.

## Methods

### Study design and population

This cross-sectional study utilized Demographic and Health Surveys (DHS) data from 45 LMICs collected between 2012 and 2022. DHS are representative household samples of the study population for each country. A stratified two-stage cluster sampling method was employed independently and at different survey periods in each country.^25^ Sampling frames were based on the respective country's population census. In the first stage, enumeration areas were selected based on household size. Then, households were chosen from clusters through systematic sampling. Household listing was conducted using tablets, and random selection was facilitated by computer programming. Interviews were conducted exclusively in pre-selected households, with no replacements or alterations allowed to prevent bias.

**Fig. 1:**
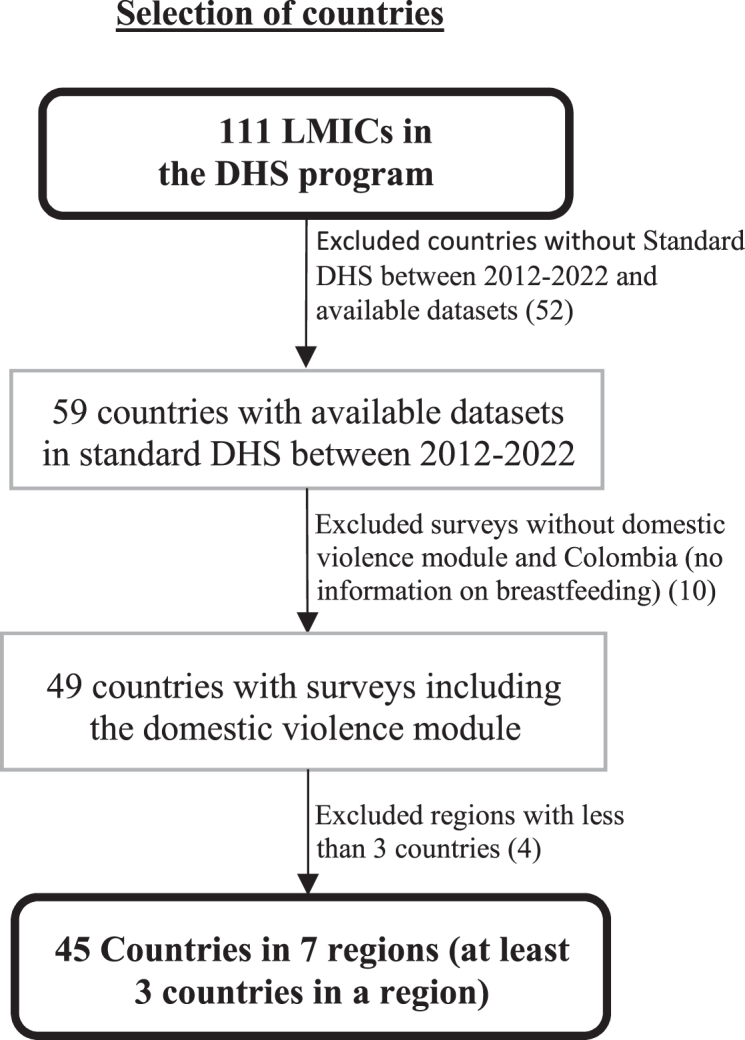
Flowchart showing the selection process of countries.

The DHS program includes nationally representative datasets from 111 LMICs across various regions. These countries were grouped into their respective regions and 45 countries were selected for this analysis. Fig. 1 illustrates the inclusion process of countries and World Bank regions for analysis, and Table 1 lists the regions, countries, and survey periods selected for this study. The datasets are mostly uniform across countries with slight adjustments for differences. Interviewers were provided with additional training on administering the questions on domestic violence and they reiterate informed consent immediately prior to administering the questions.

**Table 1:** Regions, Countries and their survey year/period.

Region	Country (Survey year/period)										
Central Africa (CA)	Cameroon (2018)	Chad (2014–2015)		Democratic Republic of Congo, DRC (2013-2014)				Gabon (2019–2021)			
East Africa (EA)	Burundi (2016–2017)	Comoros (2012)		Ethiopia (2016)	Kenya (2022)	Madagascar (2021)	Malawi (2015–2016)	Rwanda (2019–2020)		Tanzania (2022)	Uganda (2016)
Latin America & Caribbean (LA)	Dominican Republic (2013)	Guatemala (2014–2015)		Haiti (2016–2017)				Honduras (2012)		Peru (2014)	
North Africa/West Asia/Europe (NA)	Armenia (2015–2016)			Egypt (2014)				Jordan (2017–2018)			
South & Southeast Asia (SSA)	Afghanistan (2015)	Cambodia (2021–2022)		Maldives (2016–2017)		Myanmar (2015–2016)	Nepal (2022)	Pakistan 2017–2018)	Philippines (2022)	Timor-Leste (2016)	
Southern Africa (SA)	Angola (2015–2017)			Namibia (2013)		South Africa (2016)		Zambia (2018)		Zimbabwe (2015)	
West Africa (WA)	Nigeria (2018)	Benin Republic (2017–2018)	Burkina Faso (2021)	Cote d’Ivoire (2021)	Gambia (2019–2020)	Liberia (2019–2020)	Mali (2018)	Senegal (2019)	Sierra Leone (2019)	Mauritania (2019–2021)	Togo (2013–2014)

**Fig. 2:**
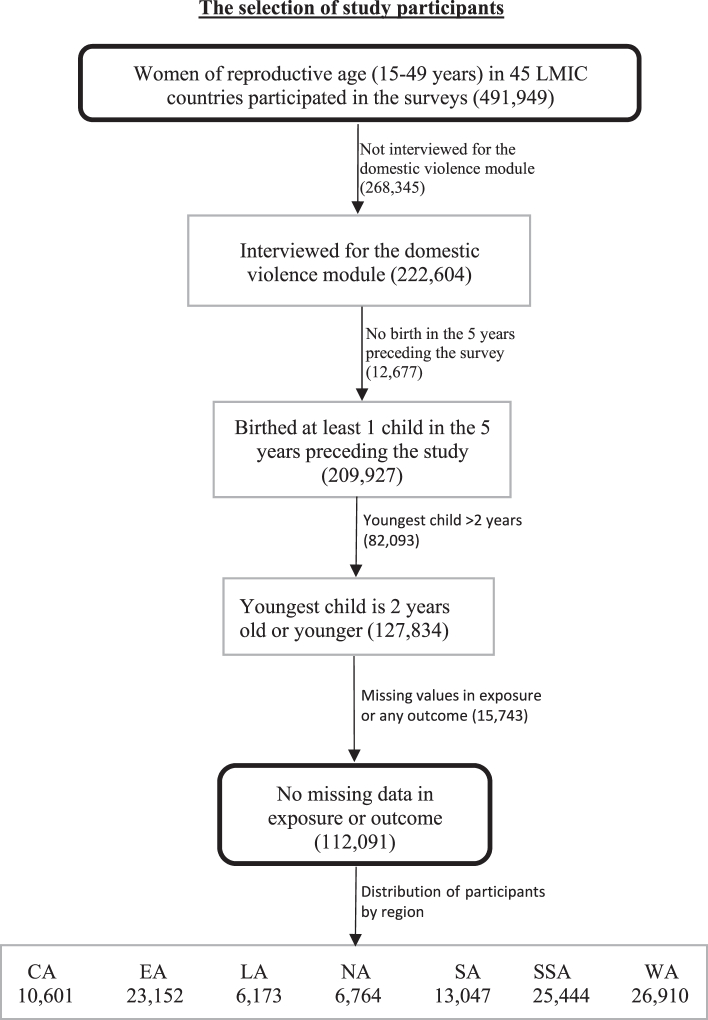
Flowchart showing the selection process of study participants.

A total of 112,091 women who were interviewed for the domestic violence module and had a child aged two years or younger were included in the analysis. The selection criteria are shown in Fig. 2. To minimize recall bias, the analysis was limited to data on the youngest child of the woman.

### Procedures

This study examined four outcomes: 1. Antenatal care utilization: Determined by at least four visits during pregnancy, coded as a binary variable (yes/no). This classification aligns with the gold standard definition of antenatal care visits provided by the WHO.^26^ Although the 2016 WHO Guidelines recommend eight antenatal care visits to reduce perinatal mortality^27^; four visits are considered adequate for this analysis based on the recommendations at the time most surveys were conducted. 2. EIBF: Defined as whether the infant was breastfed within the first hour after birth.^28^^,^^29^ This is represented as a binary variable, where “yes” indicates breastfeeding initiation within the first hour and “no” indicates otherwise. 3. EBF2D: Defined as whether a child born in the last twenty-four months was fed exclusively with breast milk for the first two days after birth.^29^ This was coded as a binary variable (yes/no) and 4. Place of delivery (POD): Coded as a binary variable with “yes” meaning delivery in a health facility (institution) and “no” meaning home delivery.^30^ All outcomes were assessed based on the most recent pregnancy, defined as the birth of the youngest child aged two years or younger.

Physical IPV during pregnancy was the main exposure variable due to the absence of measurement on other forms of violence during pregnancy in the DHS. The exposure variable, physical IPV was classified into three categories: Women never reporting physical IPV (No IPV), women reporting physical IPV only at other times (IPV at other times) and women reporting physical IPV during pregnancy (IPV during pregnancy). It is important to note that while the outcome variables refer to the most recent pregnancy, the IPV during pregnancy exposure captures experiences from any pregnancy in the woman's lifetime.

In this study, physical IPV refers to physical violence perpetrated by a partner in an intimate relationship. Respondents were asked about their life time experience of physical IPV and separately about their experiences of physical violence during pregnancy, specifying the perpetrator. Respondents who identified current or former partners as perpetrators of physical IPV during pregnancy were classified as having experienced physical IPV during pregnancy. Respondents who reported having experienced physical IPV at least once in their lifetime but not during pregnancy were classified as physical IPV at other times. Although the DHS also offers information on other forms of lifetime IPV, such as sexual and emotional, we focused on physical IPV as this was the only form measured during pregnancy for consistency.

The selection of covariates was driven primarily by an extensive review of the literature on factors known to influence both IPV and maternal/child health outcomes.^4^^,^^12^^,^^13^ These covariates include age, education level, place of residence, wealth index, media exposure, employment status, health decision-making, parity, permission needed to visit a health facility, and region were included. Age and parity were measured continuously. Education level was categorized into no education, primary, secondary, and higher based on each country's education system, place of residence as urban or rural. The household wealth index, a composite measure of living standards, was calculated based on data on selected assets and infrastructure, categorized into poorest, poor, middle, richer, and richest.^31^ Media exposure was defined as reading the newspaper, listening to the radio, or watching television at least once a week. Employment status was coded binary as yes if she was currently employed or had been on leave for the past seven days. Getting medical help for oneself was categorized as no problem, a medium problem, or a big problem. Health decision-making was categorized based on responses to who usually decided on their healthcare: woman alone, woman and partner, partner alone, someone else, and others. Countries were classified into seven regions as displayed in Table 1.^32^ Covariates were chosen to ensure that observed associations between IPV (both during and at other times) and the outcomes are not driven by underlying differences in the chosen co-variates, such as socioeconomic status, education, or access to health services.

### Ethics

The study received ethical approval from LMU Munich Medical Faculty Ethics board number 24-0437. Protocol and questionnaires for standard DHS surveys were reviewed and approved by ICF Institutional Review Board (IRB).^33^ Additionally, country-specific DHS survey protocols are reviewed by the ICF IRB and also by an IRB in the host country. Also, participants gave informed consent to participate in the study. The DHS program under the United States Agency for International Development (USAID) provided the data and granted the approval for its use in this study.

### Statistical analysis

Computational analysis was conducted using R software version 4.3.1. P-values (with a threshold of 0.05 indicating significance) and 95% confidence intervals were used to report findings. Survey weights for the domestic violence module were applied to address under- or over-sampling. Missing data for covariates were imputed using Multiple Imputation by Chained Equations. Summary statistics were determined for the overall sample and separately for each outcome. Mean and standard deviation were reported for continuous variables, while absolute frequencies and proportions were reported for categorical variables.

Univariate log-binomial regression model was used to explore the association between each variable and an outcome. Multivariable log-binomial regressions were applied to assess the association between IPV and the four behavioural outcomes of this study. The physical IPV coefficients, relative risk (RR), obtained from the models, represent the estimated differential risk in the respective outcome associated with experiencing physical IPV, controlling for the effects of other covariates in the model. For each model, the IPV variable and each covariate were added first to identify which covariate had the greatest impact on the outcome. All covariates were considered in identifying the covariate with the greatest impact. This was determined by the covariate whose model had the lowest Akaike information criterion (AIC) for an outcome. Potential multicollinearity issues among all variables in the model were investigated by computing the variance inflation factors using a threshold of five.^34^

To assess the robustness of the study findings, sensitivity analyses was performed in which the identical multivariable log-binomial regression models were re-evaluated. However, in these analyses, physical IPV was captured in two categories: 1 = “No IPV during pregnancy”, and 2 = “IPV during pregnancy”. This was done to examine whether the extra category in the initial analyses had an impact on the results.

### Role of the funding source

The funding source was not involved in the study design; in the collection, analysis, and interpretation of data; in the writing of the report; and in the decision to submit the paper for publication.

## Results

112,091 women across 45 countries who completed the domestic violence module and had a child aged 2 years or younger were initially included in the analysis. After the application of survey weights, the sample size accorded to a total of 100,199 women (mean age = 28.5 years, sd = 6.6). The regions with the highest number of study participants were West Africa (WA) and South & Southeast Asia (SSA), with 24% (24,015 of 100,199) and 23% (23,215 of 100,199) respectively. Physical IPV during pregnancy was reported by 6078 of 100,199 (6.1%, 95% CI = 5.9, 6.2) women, while 23,717 of 100,199 (23.7%, 95% CI = 23.4, 23.9) reported experiencing physical IPV at other times. Additionally, 66% (65,666 of 100,199) of the women resided in rural areas, and 36% (36,469 of 100,199) had no formal education. Further demographic details are provided in Table 2.

**Table 2:** Unadjusted summary statistics stratified by outcome variables.

Variable	Overall N = 100,199	ANC yes N = 58,303 (58.2%)	EIBF yes N = 56,056 (55.9%)	EBF2D yes N = 75,200 (75.1%)	POD yes N = 70,823 (70.7%)
**Age, mean(sd)**	28.49 (6.61)	28.52 (6.46)	28.62 (6.63)	28.50 (6.62)	28.42 (6.50)
**Physical IPV, n(%)**					
No	70,404 (70)	43,332 (62)	39,733 (56)	53,521 (76)	51,106 (73)
At other times	23,717 (24)	12,221 (52)	13,250 (56)	17,510 (74)	15,814 (67)
During pregnancy	6078 (6)	2750 (45)	3073 (51)	4169 (69)	3902 (64)
**Education, n(%)**					
No Education	36,469 (36)	13,940 (38)	19,082 (52)	25,469 (70)	19,611 (54)
Primary	29,258 (29)	17,116 (58)	18,119 (62)	23,977 (82)	20,650 (71)
Secondary	27,529 (28)	21,063 (77)	15,391 (56)	21,185 (77)	23,826 (87)
Higher	6943 (7)	6184 (89)	3464 (50)	4569 (66)	6736 (97)
**Place of residence, n(%)**					
Urban	34,533 (34)	25,197 (73)	18,422 (53)	24,999 (72)	29,870 (86)
Rural	65,666 (66)	33,106 (50)	37,634 (57)	50,201 (76)	40,953 (62)
**Wealth index, n(%)**					
Poorest	22,414 (22)	10,556 (47)	12,965 (58)	17,081 (76)	11,874 (53)
Poorer	21,409 (21)	11,391 (53)	12,095 (56)	16,396 (77)	13,286 (62)
Middle	20,850 (21)	12,087 (58)	11,733 (56)	15,660 (75)	14,837 (71)
Richer	19,103 (19)	12,132 (64)	10,459 (55)	14,389 (75)	15,647 (82)
Richest	16,423 (17)	12,137 (74)	8805 (54)	11,675 (71)	15,178 (92)
**Media exposure, n(%)**					
No	49,571 (49)	24,175 (49)	28,760 (58)	37,545 (76)	29,930 (60)
Yes	50,628 (51)	34,128 (67)	27,296 (54)	37,655 (74)	40,893 (81)
**Employment, n(%)**					
No	50,682 (51)	27,924 (55)	26,779 (53)	35,893 (71)	35,233 (70)
Yes	49,517 (49)	30,379 (61)	29,277 (59)	39,307 (79)	35,590 (72)
**Parity, mean(sd)**	3.34 (2.06)	3.06 (1.90)	3.38 (2.04)	3.34 (2.04)	3.11 (1.94)
**Health decision, n(%)**					
Woman alone	17,419 (17)	11,832 (68)	10,283 (59)	13,601 (78)	13,535 (78)
Woman and Partner	44,531 (44)	27,725 (62)	26,157 (59)	34,414 (77)	32,786 (74)
Partner alone	35,934 (36)	17,816 (50)	18,761 (52)	25,939 (72)	23,027 (64)
Someone else	1415 (2)	641 (45)	579 (41)	826 (58)	948 (67)
Other	900 (1)	290 (32)	276 (31)	421 (47)	527 (59)
**Getting medical help for self: getting permission to go, n(%)**					
No problem	1886 (2)	1612 (86)	1198 (64)	1842 (98)	1329 (70)
Big problem	22,637 (22)	9354 (41)	11,952 (53)	15,811 (70)	12,653 (56)
Not a big problem	75,676 (76)	47,337 (63)	42,906 (57)	57,547 (76)	56,841 (75)
**Region, n(%)**					
Central Africa	9398 (10)	4871 (52)	4423 (47)	7485 (80)	6150 (65)
Eastern Africa	19,738 (20)	10,513 (53)	14,400 (73)	16,812 (85)	14,588 (74)
Latin America and the Caribbean	5169 (5)	4192 (81)	2850 (55)	4404 (85)	3516 (68)
Northern Africa/Western Asia/Eastern Europe	6219 (6)	5550 (89)	2799 (45)	4782 (77)	5858 (94)
Southern Africa	12,445 (12)	8024 (64)	7356 (59)	10,967 (88)	8088 (65)
Southern & Southeast Asia	23,215 (23)	10,441 (45)	11,149 (48)	13,405 (58)	14,787 (64)
Western Africa	24,015 (24)	14,711 (61)	13,079 (54)	17,346 (72)	17,836 (74)

IPV, Intimate partner violence, ANC, Antenatal care, EIBF, Early initiation of breastfeeding, EBF2D, Exclusive breastfeeding for the first 2 days after birth, POD, Place of delivery.

Approximately 58.2% (58,303 of 100,199, 95% CI = 57.9, 58.5) of women adequately utilized antenatal care. Physical IPV during pregnancy impacted antenatal care utilization most, with 45% (2750 of 6078) experiencing physical IPV during pregnancy using it adequately, compared to 52% (12,221 of 23,717) who experienced physical IPV at other times and 62% (43,332 of 70,404) who never experienced physical IPV. Similarly, 56,056 out of 100,199 women (55.9%, 95% CI = 55.6, 56.3, mean age = 28.6 years, sd = 6.6) initiated breastfeeding within an hour of birth. In this group of women, about 51% (n = 3073 of 6078) experienced physical IPV during pregnancy, which was lower than the 56% (13,250 of 23,717) seen in women who experienced physical IPV at other times and 56% (39,733 of 70,404) also observed in women who never experienced physical IPV. Overall, 75,200 out of 100,199 women (75.1%, 95% CI = 74.8, 75.3) exclusively breastfed their child for the first two days after birth. Among women who experienced physical IPV during pregnancy, 69% (4169 of 6078) exclusively breastfed for the two days after delivery, which is a lower percentage when compared to the 74% (17,510 of 23,717) who experienced physical IPV at other times and the 76% (53,521 of 70,404) who had never experienced physical IPV. The data also revealed that about 70.7% (70,823 of 100,199, 95% CI = 74.8, 75.3) of the women delivered their babies in a health facility. In this group of women, 64% (3902 of 6078) experienced physical IPV during pregnancy, 67% (15,814 of 23,717) experienced IPV at other times, and 73% (51,106 of 70,404) never experienced physical IPV.

**Fig. 3:**
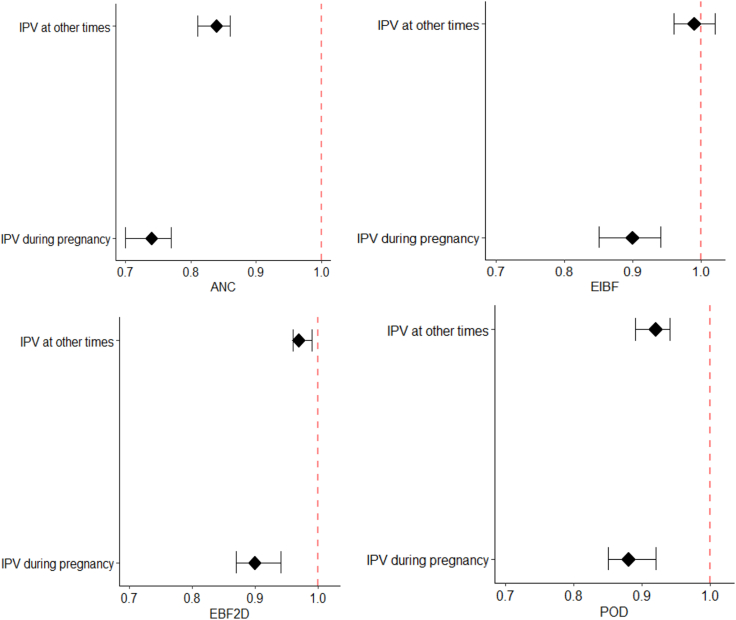
Forest plots showing the relative risk and 95% confidence intervals obtained from the univariate analysis of physical intimate partner violence (IPV) and all outcomes considered in this study which include; adequate antenatal care utilization (ANC), early initiation of breastfeeding within 1 h after birth (EIBF), exclusive breastfeeding for the first 2 days after birth (EBF2D), and place of delivery (POD).

In the univariate models, the associations between physical IPV during pregnancy and all outcomes were significant (Fig. 3 and Supplementary Table S5), except the association between EIBF and physical IPV at other times. The likelihood of adequate antenatal care utilization was lower in women who experienced physical IPV during pregnancy (RR = 0.74, 95% CI = 0.70, 0.77) and women who experienced physical IPV at other times (RR = 0.84, 95% CI = 0.81, 0.86) compared to women who never experienced physical IPV. Women who experienced physical IPV during pregnancy had a lesser likelihood of EIBF (RR = 0.90, 95% CI = 0.85, 0.94) compared to women with no experience of IPV. The experience of physical IPV during pregnancy (RR = 0.90, 95% CI = 0.87, 0.94) and at other times (RR = 0.97, 95% CI = 0.95, 0.99) had a negative impact on the likelihood of EBF2D when compared to women who never experienced physical IPV. This negative trend was also observed for POD, both in women who experienced physical IPV during pregnancy (RR = 0.88, 95% CI = 0.85, 0.92) and women who experienced physical IPV at other times (RR = 0.92, 95% CI = 0.89, 0.92).

After adjusting for all covariates, the association between physical IPV during pregnancy and antenatal care, EIBF and EBF2D remained significant. The likelihood of adequate antenatal care utilization in women who experienced physical IPV during pregnancy changed to 12% (aRR = 0.88, 95% CI = 0.84, 0.91) and 5% (aRR = 0.95, 95% CI = 0.93, 0.97) in women who experienced physical IPV at other times, when compared to women who never experienced physical IPV. Women who experienced physical IPV during pregnancy were 8% (aRR = 0.92, 95% CI = 0.89, 0.96) less likely to initiate breastfeeding early. The likelihood of EBF2D changed to 0.93 (95% CI = 0.90, 0.96) in women who experienced physical IPV during pregnancy and 0.98 (95% CI = 0.96, 0.99) in women who experienced IPV at other times when compared to women who never experienced physical IPV. The association between physical IPV and POD became insignificant in the adjusted model both in women who experienced physical IPV during pregnancy and in women who experienced physical IPV at other times. More detailed results of the models are presented in Table 3.

**Table 3:** Multivariable analyses to assess the association between IPV and the outcomes considered.

Variable	ANC		EIBF		EBF2D		POD	
	aRR	95% CI	aRR	95% CI	aRR	95% CI	aRR	95% CI
**Physical IPV**								
No	—	—	—	—	—	—	—	—
At other times	0.95∗∗∗	0.93, 0.97	1.00	0.97, 1.02	0.98∗∗	0.96, 0.99	1.00	0.98, 1.02
During pregnancy	0.88∗∗∗	0.84, 0.91	0.92∗∗∗	0.89, 0.96	0.93∗∗∗	0.90, 0.96	1.01	0.98, 1.04
**Age**	1.01∗∗∗	1.01, 1.02	1.00∗	1.00, 1.00	1.00	1.00, 1.00	1.01∗∗∗	1.01, 1.01
**Place of residence**								
Urban	—	—	—	—	—	—	—	—
Rural	0.90∗∗∗	0.87, 0.93	1.00	0.97, 1.03	1.07∗∗∗	1.04, 1.09	0.90∗∗∗	0.89, 0.92
**Education**								
No Education	—	—	—	—	—	—	—	—
Primary	1.39∗∗∗	1.35, 1.43	1.10∗∗∗	1.07, 1.12	1.05∗∗∗	1.03, 1.07	1.23∗∗∗	1.21, 1.26
Secondary	1.61∗∗∗	1.56, 1.65	1.11∗∗∗	1.08, 1.14	1.04∗∗	1.01, 1.06	1.32∗∗∗	1.29, 1.35
Higher	1.61∗∗∗	1.56, 1.67	1.04	0.99, 1.09	0.91∗∗∗	0.88, 0.95	1.28∗∗∗	1.25, 1.31
**Employment**								
No	—	—	—	—	—	—	—	—
Yes	1.09∗∗∗	1.07, 1.11	1.01	0.99, 1.04	1.04∗∗∗	1.03, 1.05	1.00	0.99, 1.02
**Wealth index**								
Poorest	—	—	—	—	—	—	—	—
Poorer	1.06∗∗∗	1.03, 1.08	0.98	0.96, 1.01	1.01	0.99, 1.03	1.12∗∗∗	1.09, 1.14
Middle	1.07∗∗∗	1.04, 1.10	0.98	0.95, 1.01	1.00	0.98, 1.03	1.21∗∗∗	1.18, 1.25
Richer	1.06∗∗	1.02, 1.10	0.95∗∗	0.92, 0.98	1.02	1.00, 1.05	1.29∗∗∗	1.26, 1.33
Richest	1.06∗	1.01, 1.11	0.93∗∗∗	0.89, 0.97	1.00	0.96, 1.03	1.30∗∗∗	1.27, 1.34
**Parity**	0.94∗∗∗	0.93, 0.95	1.00	1.00, 1.01	0.99∗	0.99, 1.00	0.95∗∗∗	0.95, 0.96
**Health decision**								
Woman alone	—	—	—	—	—	—	—	—
Woman and Partner	0.96∗∗∗	0.94, 0.97	1.01	0.99, 1.04	1.00	0.98, 1.02	0.96∗∗∗	0.95, 0.97
Partner alone	0.88∗∗∗	0.86, 0.90	0.93∗∗∗	0.90, 0.95	0.96∗∗∗	0.94, 0.98	0.92∗∗∗	0.90, 0.94
Someone else	0.89∗	0.81, 0.98	0.80∗∗∗	0.72, 0.89	0.88∗∗	0.81, 0.95	1.00	0.94, 1.06
Other	0.73∗∗	0.61, 0.88	0.62∗∗∗	0.51, 0.75	0.74∗∗	0.62, 0.89	0.92	0.80, 1.06
**Getting medical help for self: getting permission to go**								
No problem	—	—	—	—	—	—	—	—
Big problem	0.72∗∗∗	0.69, 0.76	0.80∗∗∗	0.74, 0.86	0.80∗∗∗	0.77, 0.82	0.81∗∗∗	0.76, 0.86
Not a big problem	0.87∗∗∗	0.84, 0.91	0.78∗∗∗	0.73, 0.84	0.81∗∗∗	0.79, 0.84	0.93∗∗	0.88, 0.98
**Media exposure**								
No	—	—	—	—	—	—	—	—
Yes	1.09∗∗∗	1.06, 1.11	0.96∗∗∗	0.94, 0.98	0.99	0.98, 1.01	1.11∗∗∗	1.09, 1.13
**Region**								
Central Africa	—	—	—	—	—	—	—	—
Eastern Africa	0.95∗	0.91, 0.99	1.51∗∗∗	1.44, 1.59	1.03	1.00, 1.05	1.08∗∗∗	1.04, 1.12
Latin America and the Caribbean	1.20∗∗∗	1.14, 1.25	1.05	0.98, 1.13	0.98	0.94, 1.02	0.86∗∗∗	0.81, 0.90
Northern Africa/Western Asia/Eastern Europe	1.25∗∗∗	1.20, 1.30	0.95	0.88, 1.02	1.00	0.96, 1.04	1.13∗∗∗	1.09, 1.17
Southern Africa	1.07∗∗	1.02, 1.11	1.22∗∗∗	1.15, 1.29	1.09∗∗∗	1.06, 1.12	0.89∗∗∗	0.85, 0.93
Southern & Southeast Asia	0.89∗∗∗	0.84, 0.94	1.06	0.99, 1.13	0.74∗∗∗	0.71, 0.78	0.96	0.93, 1.01
Western Africa	1.19∗∗∗	1.15, 1.24	1.19∗∗∗	1.13, 1.26	0.91∗∗∗	0.88, 0.94	1.13∗∗∗	1.09, 1.17

IPV = Intimate partner violence, ANC = Antenatal care, EIBF = Early initiation of breastfeeding, EBF2D = Exclusive breastfeeding for the first 2 days after birth, POD = Place of delivery, aRR = adjusted Risk Ratio, CI = Confidence Interval, ∗Significance at 5%: ∗P<0.05; ∗∗P<0.01; ∗∗∗P<0.001. Note that in each models (for all outcomes), all covariates were adjusted for.

Of the covariates, education level had the highest impact on the relationship between physical IPV and two outcomes: adequate antenatal care utilization and POD, as indicated by the lowest AIC value, while regional variations had the highest impact on the association between physical IPV and the outcomes EIBF, and EBF2D (Supplementary Table S3).

The sensitivity analysis, which used two categories (IPV during pregnancy vs. no IPV during pregnancy) only, did not change the direction of associations found in the multivariable models (Supplementary Table S2). The tables for the multivariable logistic regression with reported OR can be seen in Supplementary Table S4.

## Discussion

The study revealed that across 45 LMICs, physical IPV has a significant impact on maternal and child health outcomes, with the effect being more pronounced when the physical IPV occurs during pregnancy than at other times. There were notable negative associations between physical IPV during pregnancy and antenatal care utilization, EIBF, and EBF2D. Women who experienced physical IPV during pregnancy were less likely to achieve these outcomes compared to those who experienced IPV at other times or not at all, suggesting that physical IPV during pregnancy may be particularly detrimental.

The lower antenatal care utilization among women experiencing physical IPV during pregnancy may be due to several factors, including psychological stress, limited autonomy, fear of further violence, restricted mobility, shame or stigma, and access to financial resources.^35^ Pregnancy could possibly intensify existing power imbalances in relationships, increasing women's dependence on their partners and making them more vulnerable to control and abuse. These in turn make it physically and emotionally harder to access health services and subsequently antenatal care.^36^

The finding that women who experienced physical IPV during pregnancy also had lower odds of EIBF within 1 h of birth and EBF2D after birth compared to women with no experience of IPV aligns with previous research.^37^, ^38^, ^39^, ^40^ Several potential mechanisms may explain this association. Physical injuries sustained from violence during pregnancy can impair a woman's ability to EIBF and EBF2D.^41^ A woman who regularly experiences physical IPV may also be too weak to breastfeed and opt for other forms of feeding for her baby. Also, the psychological distress, emotional trauma, and lack of social support resulting from physical IPV can disrupt the mother-infant bonding process and interfere with the successful initiation and exclusive breastfeeding.^42^

The lack of association between POD and experience of physical IPV during pregnancy or at other times matches the mixed evidence base on this issue across different countries,^43^ and could be due to cross-country differences in cultural beliefs and norms about gender roles and physical IPV, the availability and quality of healthcare services, and the presence and effectiveness of regional policies and programs targeting physical IPV and promoting maternal health vary widely across regions and place of residence (urban or rural).^44^^,^^45^ These variations could affect both the prevalence of physical IPV and women's health-seeking behaviours.

In addressing the adverse effects of physical IPV during pregnancy on maternal and child health, policies should focus on training healthcare providers to recognize signs of current physical IPV during antenatal care visits and provide targeted support and interventions for affected women during pregnancy to improve health outcomes.^46^ Integrating physical IPV prevention and response strategies into maternal and child health programs is crucial to address this critical determinant of maternal and child health outcomes, particularly in LMICs where the burden of physical IPV and adverse maternal and child health outcomes is often higher.^47^ Mothers following birth require a comprehensive, continuum-of-care approach that extends well beyond the immediate postnatal period. Extending the period of care at the birthing facility could allow for more in-depth screening for IPV, as well as early identification of mothers who might be at heightened risk of ongoing health complications. This extended care could include dedicated postpartum counseling sessions, immediate mental health support, and assistance with breastfeeding initiation and maintenance. Furthermore, establishing clear referral pathways to specialized support facilities—such as domestic violence shelters, counseling centers, and community health services—can provide the necessary resources for mothers facing IPV.

Community education is needed to raise awareness about the importance of adequate antenatal care utilization and delivery in a health facility and to provide information on available support for women experiencing IPV. Policies that mandate IPV education and prevention programs in schools and communities can create a supportive environment for women to seek care, report violence, and reduce stigma.^48^ Policy recommendations should also focus on increasing demand for facility-based deliveries by addressing sociocultural, financial, and logistical barriers that prevent women from accessing health facilities. Community education, transportation support, and reducing out-of-pocket costs can help encourage safer deliveries at appropriate health facilities.^49^^,^^50^

The study's strengths include its large sample size, survey weights and the use of data from multiple LMICs, enhancing the generalizability of the findings. In addition, DHS datasets collect similar variables tailored to each country and which are uniform across different countries.

Some limitations include the cross-sectional nature of the data that prevents establishing causality. The exclusion of women with missing exposure or outcome data may have introduced systematic bias, potentially affecting the generalizability of the findings. It is important to note that matching the experience of physical IPV during pregnancy with the specific pregnancy was not possible and theoretically women could have experienced physical IPV in a different pregnancy than the one reported on. In addition, sensitive topics such as the experience of physical IPV can suffer from reporting bias which leads to under-reporting. IPV disclosure was not included as a covariate, although underreporting due to fear lead to misclassification of IPV exposure. Studies show that 20–66% of women never disclose IPV which may contribute to underestimation of its association with maternal health outcomes.^24^ Other forms of IPV including sexual IPV and emotional IPV are strongly correlated to physical IPV and were not included in the analysis.^24^ The surveys included in this study were conducted at different periods in the past and may not completely reflect the current situations in the studied countries. In future research, integrating contextual indices could help explain regional variations and offer additional insights by incorporating country level differences. This approach might involve multilevel modeling techniques to assess how these broader policy environments and cultural factors moderate the relationship between IPV and maternal and child health outcomes.

In conclusion, the pooled multivariable analysis of data from 45 LMICs reveals significant associations between physical IPV and adverse maternal and child health outcomes, with the impact being more severe when the IPV occurred during pregnancy than when it happened other times outside pregnancy. These findings are important as they highlight the far-reaching consequences of physical IPV during pregnancy on both maternal and child health outcomes. By addressing physical IPV during pregnancy, we can potentially improve not only immediate health outcomes but also long-term child development and well-being.

## Contributors

JA and HS conceptualized the study. JA curated the data. JA and NM conducted the formal analysis. JA and NM had access to the study data. NM verified the study data. HS acquired the funding. All authors were involved in the investigation and methodology. Other resources outside the external funding were provided by JA and HS. HS supervised the project. JA drafted the original manuscript with the input of all co-authors. All authors were involved in the review and editing of the manuscript and approved the final version. The corresponding author attests that all listed authors meet authorship criteria and that no other authors meeting this criteria have been excluded.

## Data sharing statement

The data used in this study is publicly available to on the DHS program website- https://dhsprogram.com/Countries/.

## Declaration of interests

We declare no competing interests.
