# Supplementary material for: Experience of physical violence during pregnancy and its association with behavioral outcomes during the prenatal and postnatal period: a pooled analysis of cross-sectional data from 45 low-income and middle-income countries
Source: eClinicalMedicine. 2025 Sep 19;89:103491. doi: 10.1016/j.eclinm.2025.103491 (PMC12495414; doi:10.1016/j.eclinm.2025.103491)
Supplement: Supplementary Tables [file mmc1.docx]

**SUPPLEMENTARY TABLES**

**Supplementary table S1; Univariate log binomial analyses to assess the association between all outcomes considered in this study and IPV and other covariates**

| **Variable** | **ANC** | | **EIBF** | | **EBF2D** | | **POD** | |
| --- | --- | --- | --- | --- | --- | --- | --- | --- |
|  | **RR** | **95% CI** | **RR** | **95% CI** | **RR** | **95% CI** | **RR** | **95% CI** |
| **Physical** **IPV** |  |  |  |  |  |  |  |  |
| No | ref | ref | ref | ref | ref | ref | ref | ref |
| At other times | 0.84*** | 0.81, 0.86 | 0.99 | 0.96, 1.02 | 0.97* | 0.95, 0.99 | 0.92*** | 0.89, 0.94 |
| During pregnancy | 0.74*** | 0.70, 0.77 | 0.90*** | 0.85, 0.94 | 0.90*** | 0.87, 0.94 | 0.88*** | 0.85, 0.92 |
| **Age** | 1.00 | 1.00, 1.00 | 1.00*** | 1.00, 1.00 | 1.00 | 1.00, 1.00 | 1.00** | 1.00, 1.00 |
| **Place of residence** |  |  |  |  |  |  |  |  |
| Urban | ref | ref | ref | ref | ref | ref | ref | ref |
| Rural | 0.69*** | 0.67, 0.71 | 1.07*** | 1.05, 1.10 | 1.06*** | 1.03, 1.08 | 0.72*** | 0.71, 0.74 |
| **Education** |  |  |  |  |  |  |  |  |
| No Education | ref | ref | ref | ref | ref | ref | ref | ref |
| Primary | 1.53*** | 1.48, 1.58 | 1.18*** | 1.15, 1.22 | 1.17*** | 1.14, 1.20 | 1.31*** | 1.28, 1.35 |
| Secondary | 2.00*** | 1.93, 2.07 | 1.07*** | 1.04, 1.10 | 1.10*** | 1.07, 1.13 | 1.61*** | 1.57, 1.65 |
| Higher | 2.33*** | 2.25, 2.41 | 0.95* | 0.91, 1.00 | 0.94** | 0.91, 0.98 | 1.80*** | 1.76, 1.85 |
| **Employment** |  |  |  |  |  |  |  |  |
| No | ref | ref | ref | ref | ref | ref | ref | ref |
| Yes | 1.11*** | 1.09, 1.14 | 1.12*** | 1.09, 1.15 | 1.12*** | 1.10, 1.14 | 1.03*** | 1.01, 1.05 |
| **Wealth index** |  |  |  |  |  |  |  |  |
| Poorest | ref | ref | ref | ref | ref | ref | ref | ref |
| Poorer | 1.13*** | 1.10, 1.16 | 0.98 | 0.95, 1.00 | 1.00 | 0.99, 1.02 | 1.17*** | 1.14, 1.20 |
| Middle | 1.23*** | 1.19, 1.28 | 0.97 | 0.94, 1.01 | 0.99 | 0.96, 1.01 | 1.34*** | 1.30, 1.39 |
| Richer | 1.35*** | 1.30, 1.40 | 0.95*** | 0.92, 0.98 | 0.99 | 0.97, 1.01 | 1.55*** | 1.50, 1.59 |
| Richest | 1.57*** | 1.51, 1.63 | 0.93*** | 0.89, 0.96 | 0.93*** | 0.90, 0.96 | 1.74*** | 1.70, 1.79 |
| **Parity** | 0.93*** | 0.93, 0.93 | 1.01*** | 1.01, 1.01 | 1.00 | 1.00, 1.00 | 0.94*** | 0.94, 0.95 |
| **Health decision** |  |  |  |  |  |  |  |  |
| Woman alone | ref | ref | ref | ref | ref | ref | ref | ref |
| Woman and Partner | 0.92*** | 0.90, 0.93 | 1.0 | 0.97, 1.02 | 0.99 | 0.97, 1.01 | 0.95*** | 0.93, 0.96 |
| Partner alone | 0.73*** | 0.71, 0.75 | 0.88*** | 0.86, 0.91 | 0.92*** | 0.90, 0.95 | 0.82*** | 0.81, 0.84 |
| Someone else | 0.67*** | 0.60, 0.75 | 0.69*** | 0.62, 0.77 | 0.75*** | 0.69, 0.82 | 0.86*** | 0.81, 0.92 |
| Other | 0.47*** | 0.37, 0.61 | 0.52*** | 0.42, 0.64 | 0.60*** | 0.49, 0.74 | 0.75** | 0.63, 0.91 |
| **Getting medical help for self: getting permission to go** |  |  |  |  |  |  |  |  |
| No problem | ref | ref | ref | ref | ref | ref | ref | ref |
| Big problem | 0.48*** | 0.46, 0.51 | 0.83*** | 0.78, 0.88 | 0.71*** | 0.70, 0.73 | 0.79*** | 0.75, 0.84 |
| Not a big problem | 0.73*** | 0.71, 0.75 | 0.89*** | 0.85, 0.94 | 0.78*** | 0.77, 0.79 | 1.07* | 1.01, 1.12 |
| **Media exposure** |  |  |  |  |  |  |  |  |
| No | ref | ref | ref | ref | ref | ref | ref | ref |
| Yes | 1.38*** | 1.35, 1.42 | 0.93*** | 0.91, 0.95 | 0.98* | 0.97, 1.00 | 1.34*** | 1.32, 1.36 |
| **Region** |  |  |  |  |  |  |  |  |
| Central Africa | ref | ref | ref | ref | ref | ref | ref | ref |
| East Africa | 1.03 | 0.98, 1.08 | 1.55*** | 1.48, 1.63 | 1.07*** | 1.04, 1.10 | 1.13*** | 1.08, 1.18 |
| Latin America | 1.56*** | 1.50, 1.64 | 1.17*** | 1.10, 1.24 | 1.07*** | 1.04, 1.10 | 1.04 | 0.99, 1.10 |
| North Africa/ West Asia/ East Europe | 1.72*** | 1.65, 1.80 | 0.96 | 0.89, 1.02 | 0.97* | 0.93, 1.00 | 1.44*** | 1.38, 1.50 |
| Southern Africa | 1.24*** | 1.18, 1.31 | 1.26*** | 1.19, 1.33 | 1.11*** | 1.08, 1.14 | 0.99 | 0.94, 1.05 |
| South & Southeast Asia | 0.87*** | 0.81, 0.93 | 1.02 | 0.96, 1.09 | 0.73*** | 0.69, 0.76 | 0.97 | 0.92, 1.03 |
| West Africa | 1.18*** | 1.13, 1.23 | 1.16*** | 1.10, 1.22 | 0.91*** | 0.88, 0.93 | 1.13*** | 1.09, 1.18 |

IPV= Intimate partner violence, ANC= Antenatal care, EIBF= Early initiation of breastfeeding, EBF2D= Exclusive breastfeeding for the first 2 days after birth, POD= Place of delivery, RR=Relative Risk, CI = Confidence Interval, *Significance at 5%: *p<0.05; **p<0.01; ***p<0.001

Note that in each models (for all outcomes), all covariates were adjusted for.

ANC= Antenatal care, EIBF= Early initiation of breastfeeding, EBF2D= Exclusive breastfeeding for the first 2 days after birth, POD= Place of delivery

**Supplementary table S2; Multivariable log-binomial regression to assess the robustness of study findings in the association between IPV during pregnancy (2 categories) and all considered outcomes**

| **Variable** | **ANC** | | **EIBF** | | **EBF2D** | | **POD** | |
| --- | --- | --- | --- | --- | --- | --- | --- | --- |
|  | **aRR** | **95% CI** | **aRR** | **95% CI** | **aRR** | **95% CI** | **aRR** | **95% CI** |
| **Physical** **IPV during pregnancy** |  |  |  |  |  |  |  |  |
| No | ref | ref | ref | ref | ref | ref | ref | ref |
| Yes | 0.89*** | 0.86, 0.92 | 0.93*** | 0.89, 0.96 | 0.93*** | 0.91, 0.96 | 1.01 | 0.98, 1.03 |
| **Age** | 1.01*** | 1.01, 1.02 | 1.00* | 1.00, 1.00 | 1.00 | 1.00, 1.00 | 1.01*** | 1.01, 1.01 |
| **Place of residence** |  |  |  |  |  |  |  |  |
| Urban | ref | ref | ref | ref | ref | ref | ref | ref |
| Rural | 0.90*** | 0.87, 0.93 | 1.00 | 0.97, 1.03 | 1.07*** | 1.04, 1.09 | 0.90*** | 0.89, 0.92 |
| **Education** |  |  |  |  |  |  |  |  |
| No Education | ref | ref | ref | ref | ref | ref | ref | ref |
| Primary | 1.39*** | 1.35, 1.43 | 1.10*** | 1.07, 1.12 | 1.05*** | 1.03, 1.07 | 1.23*** | 1.21, 1.26 |
| Secondary | 1.61*** | 1.56, 1.66 | 1.11*** | 1.08, 1.14 | 1.04** | 1.01, 1.06 | 1.32*** | 1.29, 1.35 |
| Higher | 1.62*** | 1.57, 1.68 | 1.04 | 0.99, 1.09 | 0.91*** | 0.88, 0.95 | 1.28*** | 1.25, 1.31 |
| **Employment** |  |  |  |  |  |  |  |  |
| No | ref | ref | ref | ref | ref | ref | ref | ref |
| Yes | 1.09*** | 1.07, 1.10 | 1.01 | 0.99, 1.04 | 1.04*** | 1.02, 1.05 | 1.00 | 0.99, 1.02 |
| **Wealth index** |  |  |  |  |  |  |  |  |
| Poorest | ref | ref | ref | ref | ref | ref | ref | ref |
| Poorer | 1.06*** | 1.03, 1.08 | 0.98 | 0.96, 1.01 | 1.01 | 0.99, 1.03 | 1.12*** | 1.09, 1.14 |
| Middle | 1.07*** | 1.04, 1.10 | 0.98 | 0.95, 1.01 | 1.00 | 0.98, 1.03 | 1.21*** | 1.18, 1.25 |
| Richer | 1.06** | 1.02, 1.10 | 0.95** | 0.92, 0.98 | 1.02 | 1.00, 1.05 | 1.29*** | 1.26, 1.33 |
| Richest | 1.06* | 1.01, 1.11 | 0.93*** | 0.89, 0.97 | 1.00 | 0.96, 1.04 | 1.30*** | 1.27, 1.34 |
| **Parity** | 0.94*** | 0.93, 0.95 | 1.00 | 1.00, 1.01 | 0.99* | 0.99, 1.00 | 0.95*** | 0.95, 0.96 |
| **Health decision** |  |  |  |  |  |  |  |  |
| Woman alone | ref | ref | ref | ref | ref | ref | ref | ref |
| Woman and Partner | 0.96*** | 0.94, 0.98 | 1.01 | 0.99, 1.04 | 1.00 | 0.98, 1.02 | 0.96*** | 0.95, 0.97 |
| Partner alone | 0.88*** | 0.86, 0.90 | 0.93*** | 0.90, 0.95 | 0.96*** | 0.94, 0.98 | 0.92*** | 0.90, 0.94 |
| Someone else | 0.89* | 0.81, 0.98 | 0.80*** | 0.72, 0.89 | 0.88** | 0.81, 0.95 | 1.00 | 0.94, 1.06 |
| Other | 0.73** | 0.60, 0.88 | 0.62*** | 0.51, 0.75 | 0.74** | 0.62, 0.89 | 0.92 | 0.80, 1.07 |
| **Getting medical help for self: getting permission to go** |  |  |  |  |  |  |  |  |
| No problem | ref | ref | ref | ref | ref | ref | ref | ref |
| Big problem | 0.72*** | 0.68, 0.75 | 0.80*** | 0.74, 0.86 | 0.80*** | 0.77, 0.82 | 0.81*** | 0.76, 0.86 |
| Not a big problem | 0.87*** | 0.84, 0.91 | 0.78*** | 0.73, 0.84 | 0.81*** | 0.79, 0.84 | 0.93** | 0.88, 0.98 |
| **Media exposure** |  |  |  |  |  |  |  |  |
| No | ref | ref | ref | ref | ref | ref | ref | ref |
| Yes | 1.09*** | 1.06, 1.11 | 0.96*** | 0.94, 0.98 | 0.99 | 0.98, 1.01 | 1.11*** | 1.09, 1.13 |
| **Region** |  |  |  |  |  |  |  |  |
| Central Africa | ref | ref | ref | ref | ref | ref | ref | ref |
| Eastern Africa | 0.95* | 0.91, 0.99 | 1.51*** | 1.44, 1.59 | 1.03 | 1.00, 1.06 | 1.08*** | 1.04, 1.12 |
| Latin America and the Caribbean | 1.20*** | 1.15, 1.26 | 1.05 | 0.98, 1.13 | 0.98 | 0.94, 1.02 | 0.86*** | 0.81, 0.90 |
| Northern Africa/ Western Asia/ Eastern Europe | 1.26*** | 1.21, 1.31 | 0.95 | 0.88, 1.02 | 1.00 | 0.96, 1.04 | 1.13*** | 1.09, 1.17 |
| Southern Africa | 1.07** | 1.03, 1.12 | 1.22*** | 1.15, 1.29 | 1.09*** | 1.06, 1.12 | 0.89*** | 0.85, 0.93 |
| Southern & Southeast Asia | 0.89*** | 0.84, 0.95 | 1.06 | 0.99, 1.13 | 0.75*** | 0.71, 0.78 | 0.96 | 0.93, 1.01 |
| Western Africa | 1.20*** | 1.16, 1.25 | 1.19*** | 1.13, 1.26 | 0.91*** | 0.89, 0.94 | 1.13*** | 1.09, 1.17 |
| IPV= Intimate partner violence, ANC= Antenatal care, EIBF= Early initiation of breastfeeding, EBF2D= Exclusive breastfeeding for the first 2 days after birth, POD= Place of delivery, aRR= adjustedRelative Risk, CI = Confidence Interval, *Significance at 5%: *p<0.05; **p<0.01; ***p<0.001 | | | | | | | | |

**Supplementary table S3; Table showing the AICs of models containing the IPV variable and each individual covariate**

| **Variable** | **Akaike Information Criterion (AIC)** | | | |
| --- | --- | --- | --- | --- |
|  | **ANC** | **EIBF** | **EBF2D** | **POD** |
| **Age** | 70073.8 | 72788.2 | 48243.4 | 54839.0 |
| **Education** | 64537.4 | 72467.3 | 47788.2 | 51465.8 |
| **Place of residence** | 68079.1 | 72739.0 | 48182.3 | 52888.6 |
| **Wealth index** | 68706.0 | 72779.7 | 48194.6 | 51958.1 |
| **Media exposure** | 68460.9 | 72727.9 | 48236.7 | 53223.9 |
| **Employment** | 69883.1 | 72617.4 | 47969.8 | 54825.6 |
| **Parity** | 68899.7 | 72785.5 | 48243.0 | 53826.6 |
| **Health decision** | 69025.7 | 72428.4 | 47987.3 | 54418.0 |
| **Getting medical help for self** | 68387.1 | 72749.3 | 48025.9 | 53840.0 |
| **Region** | 67737.5 | 71151.6 | 46430.7 | 54064.3 |

**Supplementary table S4; Multivariable logistic regression with odds ratio reported**

| **Variable** | **ANC** | | **EIBF** | | **EBF2D** | | **POD** | |
| --- | --- | --- | --- | --- | --- | --- | --- | --- |
|  | **aOR** | **95% CI** | **aOR** | **95% CI** | **aOR** | **95% CI** | **aOR** | **95% CI** |
| **Physical** **IPV** |  |  |  |  |  |  |  |  |
| No | ref | ref | ref | ref | ref | ref | ref | ref |
| Other Times | 0.88*** | 0.83, 0.92 | 0.99 | 0.94, 1.05 | 0.90** | 0.84, 0.97 | 0.99 | 0.93, 1.06 |
| During pregnancy | 0.76*** | 0.69, 0.83 | 0.84*** | 0.77, 0.92 | 0.75** | 0.68, 0.84 | 1.07 | 0.98, 1.18 |
| **Age** | 1.04*** | 1.04, 1.05 | 1.00* | 1.00, 1.01 | 1.01 | 1.00, 1.01 | 1.04*** | 1.04, 1.05 |
| **Place of residence** |  |  |  |  |  |  |  |  |
| Urban | ref | ref | ref | ref | ref | ref | ref | ref |
| Rural | 0.72*** | 0.65, 0.81 | 1.00 | 0.94, 1.07 | 1.33*** | 1.19, 1.48 | 0.63*** | 0.57, 0.69 |
| **Education** |  |  |  |  |  |  |  |  |
| No Education | ref | ref | ref | ref | ref | ref | ref | ref |
| Primary | 1.86*** | 1.75, 1.97 | 1.24*** | 1.17, 1.31 | 1.25*** | 1.16, 1.35 | 1.83*** | 1.73, 1.95 |
| Secondary | 3.12*** | 2.91, 3.35 | 1.25*** | 1.17, 1.34 | 1.13** | 1.04, 1.24 | 2.93*** | 2.70, 3.17 |
| Higher | 5.02*** | 4.35, 5.79 | 1.08 | 0.97, 1.20 | 0.70*** | 0.62, 0.80 | 7.07*** | 5.73, 8.72 |
| **Employment** |  |  |  |  |  |  |  |  |
| No | ref | ref | ref | ref | ref | ref | ref | ref |
| Yes | 1.27*** | 1.21, 1.34 | 1.03 | 0.98, 1.09 | 1.18*** | 1.11, 1.26 | 1.01 | 0.95, 1.07 |
| **Wealth index** |  |  |  |  |  |  |  |  |
| Poorest | ref | ref | ref | ref | ref | ref | ref | ref |
| Poorer | 1.14*** | 1.08, 1.21 | 0.96 | 0.91, 1.02 | 1.05 | 0.97, 1.13 | 1.28*** | 1.20, 1.36 |
| Middle | 1.19*** | 1.11, 1.28 | 0.96 | 0.89, 1.04 | 1.02 | 0.92, 1.13 | 1.65*** | 1.53, 1.79 |
| Richer | 1.20*** | 1.10, 1.31 | 0.89** | 0.83, 0.96 | 1.11* | 1.00, 1.23 | 2.38*** | 2.16, 2.62 |
| Richest | 1.27** | 1.10, 1.47 | 0.84*** | 0.77, 0.93 | 1.02 | 0.87, 1.18 | 3.81*** | 3.30, 4.39 |
| **Parity** | 0.85*** | 0.83, 0.86 | 1.01 | 1.00, 1.03 | 0.98 | 0.96, 1.00 | 0.83*** | 0.82, 0.85 |
| **Health decision** |  |  |  |  |  |  |  |  |
| Woman alone | ref | ref | ref | ref | ref | ref | ref | ref |
| Woman and Partner | 0.88*** | 0.83, 0.93 | 1.03 | 0.98, 1.09 | 1.00 | 0.93, 1.09 | 0.85*** | 0.79, 0.91 |
| Partner alone | 0.72*** | 0.67, 0.77 | 0.84*** | 0.79, 0.90 | 0.84*** | 0.77, 0.91 | 0.72*** | 0.67, 0.77 |
| Someone else | 0.74** | 0.60, 0.92 | 0.65*** | 0.54, 0.79 | 0.68*** | 0.55, 0.85 | 0.94 | 0.75, 1.16 |
| Other | 0.56*** | 0.41, 0.77 | 0.44*** | 0.34, 0.59 | 0.49*** | 0.35, 0.69 | 0.75 | 0.50, 1.12 |
| **Getting medical help for self: getting permission to go** |  |  |  |  |  |  |  |  |
| No problem | ref — | ref | ref | ref | ref | ref | ref | ref |
| Big problem | 0.34*** | 0.27, 0.44 | 0.59*** | 0.50, 0.70 | 0.08*** | 0.06, 0.12 | 0.45*** | 0.36, 0.56 |
| Not a big problem | 0.51*** | 0.40, 0.65 | 0.57*** | 0.48, 0.67 | 0.09*** | 0.06, 0.13 | 0.67*** | 0.54, 0.83 |
| **Media exposure** |  |  |  |  |  |  |  |  |
| No | ref | ref | ref | ref | ref | ref | ref | ref |
| Yes | 1.22*** | 1.15, 1.28 | 0.90*** | 0.86, 0.95 | 0.96 | 0.90, 1.03 | 1.47*** | 1.38, 1.55 |
| **Region** |  |  |  |  |  |  |  |  |
| Central Africa | ref | ref | ref | ref | ref | ref | ref | ref |
| East Africa | 0.89* | 0.80, 0.99 | 2.88*** | 2.59, 3.21 | 1.23** | 1.07, 1.42 | 1.37*** | 1.19, 1.57 |
| Latin America | 2.16*** | 1.81, 2.57 | 1.10 | 0.96, 1.26 | 0.90 | 0.75, 1.09 | 0.50*** | 0.41, 0.61 |
| North Africa/ West Asia/ East Europe | 3.41*** | 2.90, 4.00 | 0.91 | 0.79, 1.04 | 0.98 | 0.82, 1.18 | 3.00*** | 2.42, 3.71 |
| Southern Africa | 1.23*** | 1.10, 1.37 | 1.53*** | 1.35, 1.72 | 1.78*** | 1.52, 2.08 | 0.64*** | 0.55, 0.74 |
| South & Southeast Asia | 0.78*** | 0.68, 0.90 | 1.13 | 1.00, 1.27 | 0.38*** | 0.32, 0.45 | 0.85* | 0.74, 0.98 |
| West Africa | 1.59*** | 1.45, 1.76 | 1.45*** | 1.31, 1.60 | 0.67*** | 0.58, 0.76 | 1.69*** | 1.48, 1.93 |

IPV = Intimate partner violence, ANC = Antenatal care, EIBF = Early initiation of breastfeeding, EBF2D = Exclusive breastfeeding for the first 2 days after birth, POD = Place of delivery, aOR = Adjusted Odds Ratio, CI = Confidence Interval, *Significance at 5%: *p<0.05; **p<0.01; ***p<0.001

**Supplementary table S5; Table showing the Variance inflation factors of the four main multivariable log binomial models**

| **Variable** | **Variance Inflation Factor (VIF)** | | | |
| --- | --- | --- | --- | --- |
|  | **ANC** | **EIBF** | **EBF2D** | **POD** |
| **Physical** **IPV** | 1.054 | 1.073 | 1.142 | 1.107 |
| **Age** | 1.857 | 1.647 | 2.105 | 1.594 |
| **Place of residence** | 1.880 | 1.226 | 1.666 | 1.304 |
| **Education** | 1.179 | 1.156 | 1.202 | 1.163 |
| **Employment** | 1.182 | 1.129 | 1.273 | 1.178 |
| **Wealth index** | 1.198 | 1.118 | 1.206 | 1.137 |
| **Parity** | 1.865 | 1.652 | 2.013 | 1.630 |
| **Health decision** | 1.081 | 1.081 | 1.162 | 1.101 |
| **Getting medical help for self** | 1.234 | 1.193 | 1.840 | 1.228 |
| **Media exposure** | 1.229 | 1.095 | 1.298 | 1.084 |
| **Region** | 1.239 | 1.143 | 1.391 | 1.184 |

IPV = Intimate partner violence, ANC = Antenatal care, EIBF = Early initiation of breastfeeding, EBF2D = Exclusive breastfeeding for the first 2 days after birth, POD = Place of delivery

**Supplementary table S6; Number of participants with missing data in each variable before missing data imputation** **and description of multiple imputation process**

| **S/N** | **Variable** | **Number of participants** |
| --- | --- | --- |
| 1. | Employment | 10 |
| 2. | Health decision | 5,913 |
| 3. | Getting medical help for self | 77 |
| 4. | Media Exposure | 72 |

Imputation Model Specification:
 – Multiple Imputation by Chained Equations procedure implemented in R (using the ‘mice’ package) was used.
 – All variables included in the main analysis (age, education, place of residence, wealth index, media exposure, employment status, health decision-making, parity, permission needed to visit a health facility, and region) were incorporated into the imputation model.
 – The imputation method was tailored to the type of variable: predictive mean matching (PMM) was used for continuous variables, logistic regression for binary variables, and multinomial logistic regression for categorical variables with more than two levels.

• Imputation Process:
 – Five imputed datasets were generated to ensure stable estimates.
 – Each dataset was created by iteratively cycling through the variables with missing data and imputing values based on regression models conditioned on the other variables.
